# Detection of Cadmium Risk to the Photosynthetic Performance of *Hybrid Pennisetum*

**DOI:** 10.3389/fpls.2019.00798

**Published:** 2019-06-20

**Authors:** Xiliang Song, Xian Yue, Weifeng Chen, Huixin Jiang, Yanyun Han, Xu Li

**Affiliations:** ^1^College of Resources and Environment, Shandong Agricultural University, Tai’an, China; ^2^Shandong Provincial Engineering and Technology Research Center for Phyto-Microremediation in Saline-Alkali Land, Shandong, China; ^3^Shandong Provincial Animal Husbandry General Station, Shandong Province Grass Products Quality Inspection Center, Jinan, China

**Keywords:** cadmium stress, photosynthetic performance, electron transport, non-stomatal limitation, *Hybrid Pennisetum*

## Abstract

Photosynthesis plays an essential role in plant growth and crop yield, and the mechanisms of the effects of cadmium (Cd) on photosynthetic performance require more attention. The acute toxicity of Cd in soil to the photosynthetic capacity of *Hybrid Pennisetum* was evaluated using gas exchange parameters, *A*/*C_i_* curves, light response curves, and chlorophyll *a* fluorescence transients after exposure to elevated Cd concentrations (0, 10, 20, 50, 70, and 100 mg kg^−1^) for a 3-month period. The results indicated that leaf Cd concentration in *Hybrid Pennisetum* increased with the strength of soil Cd stress and ranged from 4.9 to 15.8 μg g^−1^ DW. The accumulation of leaf Cd severely restricted photosynthesis and its non-stomatal limitation in regulating the photosynthetic performance of *Hybrid Pennisetum*. The leaf chloroplasts at 10 and 20 mg kg^−1^ Cd concentrations showed no noticeable change, but the chlorophyll content significantly decreased by 9.0–20.4% at 50–100 mg kg^−1^ Cd concentrations. The Cd treatments also decreased plant ribulose-1,5-bisphosphate (RuBP) activity (*V_cmax_*) and regeneration capacity (*J_max_*), triose phosphate utilization (*TPU*), light-saturated photosynthesis (*A_max_*), apparent quantum yield (*AQY*), light saturation point (*LSP*), and dark respiration (*R_day_*), but Cd treatment increased the light compensation point (*LCP*). The shape of chlorophyll *a* fluorescence transients in leaves was altered under different Cd treatments. The increased OJ phase and the decreased IP phase in fluorescence induction curves suggested that Cd toxicity inhibited both light use efficiency and photodamage avoidance ability. These results suggested that the decrease in photosynthesis through exposure to Cd may be a result of the decrease in leaf chlorophyll content, Rubisco activity, and RuBP regeneration, inhibition of triose phosphate utilization, reduction of the ability to use light and provide energy, and restrictions on electron transport in PSII.

## Introduction

With the increasing anthropogenic activities of phosphate fertilizer abuse and sewage sludge, herbicide and pesticide application, excessive industrial and aquaculture wastewater for being used for irrigation, as well as a high frequency of mining and smelting, contaminated soil polluted by heavy metals is attracting increasing attention worldwide (Mcintyre, 2003; Rizwan et al., 2017). Cadmium (Cd), as one of the most harmful heavy metals in soils, has been widely accepted to be an extremely dangerous pollutant due to its acute toxicity, high water solubility, non-degradability, and persistence inside most live organisms (Ghosh and Singh, 2005; Groppa et al., 2012; Parmar et al., 2013). Studies have found that Cd can be more easily taken up by many plant species than most other heavy metals (Gallego et al., 2012; Yu et al., 2017). Excessive accumulation of Cd^2+^ in plant tissues could cause serious phytotoxicity (Groppa et al., 2007) and numerous morphological, physiological, and biochemical toxic effects on plant growth and development (Lutts and Lefèvre, 2015; Wali et al., 2015), such as destroying leaf chlorophyll structure (Santos et al., 2018), depressing photosynthesis and respiration (Hendrik et al., 2010; Lee et al., 2010; Zhang et al., 2015), disturbing the uptake and translocation of mineral nutrients (Erdal and Turk, 2016), accumulating reactive oxygen species (ROS) (Gallego et al., 2012), restraining protein synthesis and enzymatic activity (Fornazier, 2002; Wu et al., 2015), dwarfing plants (Rascio et al., 2008), inhibiting the growth of roots (Vartika et al., 2005), decreasing biomass (Rascio et al., 2008), and even leading to plant death if Cd exceeds their resistance values (Clemens et al., 2013; Shanying et al., 2017).

Among all the physiological and biochemical processes in plants, photosynthesis, as a central carbon anabolic pathway providing energy-rich organic compounds, plays an essential role in maintaining the equilibrium between the light energy absorbed by photosystems and the chemical energy consumed by metabolic sinks (Brestic et al., 2015). The efficiency of photosynthesis has a direct relationship with plant growth and development (Kalaji et al., 2017). Photosynthesis is also a process that is highly sensitive to any changes in environmental conditions. Actually, it has been proven to be a primary target of inhibition caused by metal-induced stress (Joshi and Mohanty, 2004). Any structural and functional alterations in the photosynthetic apparatus will adversely affect physiological activities and likely affect plant growth and survival (Baek et al., 2004). Therefore, a deep understanding of the responses of plant photosynthetic performance to heavy metal stress is necessary to scientifically address the problems of soil contamination.

The deleterious effects of Cd stress on photosynthetic attributes occur in various ways. Cd stress inhibits the biosynthesis of chlorophylls (Stephen et al., 2007; Shukla et al., 2008) and their stable binding to proteins (Horváth et al., 1996), causing stomatal opening (Sandalio et al., 2001), damaging light-harvesting complex II and photosystems (Hendrik et al., 2010), decreasing the transcription of photosynthesis-related genes such as psbA, psaB, and rbcL (Qian et al., 2009), thereby inducing a considerable reduction in the quantum yields of both photosystem I (PSI) and photosystem II (PSII) and the electron transport rate and slowing down the net photosynthetic rate (Goussi et al., 2018). PSII of photosynthesis is the primary target of Cd toxicity (Alexander et al., 2008; Popova et al., 2009). Cadmium may arrest the photosynthetic electron flow (Voigt and Nagel, 2002), inhibit the water-splitting complex of the oxidizing site of PS II (Nirupama and Mohn, 2003), or competitively bind to the essential Ca^2+^ site in PSII during photoactivation (Faller et al., 2005). Furthermore, Cd has also been shown to inhibit energy utilization and carbon sequestration by slowing down the activity of various enzymes, such as ribulose-1,5-bisphosphate (RuBP) carboxylase oxygenase (Mobin and Khan, 2007), phosphoenolpyruvate carboxylase (Alexander et al., 2008), aldolase (Sheoran et al., 1990), fructose-6-phosphate kinase (Malik et al., 1992), fructose-1,6-bisphosphatase (Sheoran et al., 1990), NADP^+^-glyceraldehyde-3-phosphate dehydrogenase (Sheoran et al., 1990), and carbonic anhydrase (Mobin and Khan, 2007).

Although great progress has been made in understanding the Cd hazard to plant photosynthetic performance (Li et al., 2013), the effects of Cd ions on photosynthetic performance varied with different Cd concentrations, plant species, and stress tolerance degrees (Ghnaya et al., 2005; Mobin and Khan, 2007; Wali et al., 2016, 2017). Therefore, the disturbance mechanisms of Cd on photosynthesis still need to be studied further. *Hybrid Pennisetum* is a high-stalk perennial grass and is widely planted in south China for the remarkable advantages in fast growth, high biomass, and high ability in endure adverse stress conditions. However, the Cd risk to *Hybrid Pennisetum* has seldom been explored. The aims of this work were to explore the changes in photosystem efficiency and photosynthetic activity in the leaves of *Hybrid Pennisetum* treated with different amounts of Cd. In the present study, leaf gas exchange parameters, light response curves, CO_2_ response curves, and chlorophyll *a* fluorescence transients were determined to examine the photosynthetic responses of *Hybrid Pennisetum* to soil Cd stress.

## Materials and Methods

### Plant Material and Growth Conditions

The pot experiment was conducted in a greenhouse at the Experimental Station of Shandong Agricultural University, Tai’an, China (36°09′N, 117°09′E). Growing conditions in the greenhouse were maintained as follows: 27/22°C temperature (day/night), a 14 h photoperiod per day, 65–75% relative humidity, and 1000 mol⋅m^−2^ day^−1^ of average daily photosynthetically active radiation (PAR).

The seeds of *Hybrid Pennisetum* (cv. Ningza NO. 4), kindly provided by Shandong Provincial Animal Husbandry General Station, were used in this study. Healthy seeds were surface-sterilized in 10% H_2_O_2_ for 10 min and cleaned with sterile deionized water three times. Seeds were then sown in plastic pots (8 cm in height, 10 cm in diameter) containing soil composed of peat and vermiculite (4:1, w/w) mixed with sand (3:1, w/w) for germination.

### Experimental Designs

Six treatments (0, 10, 20, 50, 70, and 100 mg⋅kg^−1^ Cd) with four replicates were set up and designated as Cd0, Cd10, Cd20, Cd50, Cd70, and Cd100, respectively. Each plastic pot (40 cm in height and 48 cm in diameter) was filled with 20.0 kg of air-dried soil (sieved through 2 cm mesh) that was retrieved from the local surface soil (0–20 cm) and then saturated with a heavy metal solution containing the required amount of CdCl_2_⋅2.5H_2_O. The soil basic physical and chemical properties were as follows: soil texture of 73.8% sand, 12.1% silt, and 14.1% clay, soil pH of 6.67, organic matter of 13.52 g kg^−1^, available Cd concentration of 0.08 mg kg^−1^, total nitrogen of 75.62 mg kg^−1^, available phosphorus of 67.38 mg kg^−1^, and available potassium of 80.12 mg kg^−1^. After the third leaf emerged (approximately 2 weeks after sowing), five uniform seedlings were transferred to Cd-treated plastic pots. All pots were watered each day to keep the soil moisture at 75–85% with deionized water. To avoid nutrient deficiency, the soil was supplied with urea (0.20 g N kg^−1^), diammonium phosphate (0.15 g P_2_O_5_ kg^−1^) and potassium sulfate (0.20 g K_2_O kg^−1^) as basal fertilizers in each pot. All the following measurements were conducted after three months of plant growth with Cd treatments. Each plant was tested starting from the treatment of Cd0–Cd100 and again starting from the treatment of Cd0 to reduce the differences caused by the determination time.

### Chlorophyll Content Measurements

The SPAD values of chlorophyll were determined with a portable chlorophyll meter (SPAD-502, Osaka 590-8551, Japan). Leaf samples were obtained from new and fully expanded leaves from the tip, with 15 plant replicates for each treatment.

### Gas Exchange Measurements

The second fully expanded young leaves from the tops of the plants were used to estimate the net photosynthetic rate (*P_n_*), stomatal conductance (*G_s_*), intercellular CO_2_ concentration (*C_i_*), transpiration rate (*T_r_*), and water use efficiency (*WUE* = *P_n_*/*T_r_*) using a portable photosynthesis system (CIRAS-3, PP-system, Hitchin, United Kingdom). The experiment was performed on sunny days from 9:00 to 11:00 am and 14:00 to 16:00 pm. The illumination was supplied to the leaves from a red-blue LED light source. Leaf chamber temperature was maintained at room temperature with a CO_2_ concentration of 390 ppm and a photosynthetic photon flux density (PPFD) of 1000 mol photon⋅m^−2^⋅s^−1^. These measurements were performed on three plants in each Cd treatment.

### Light Response Curves

Light response curves were measured in the same leaf that was chosen for gas exchange parameter measurement with a portable photosynthesis system (CIRAS-3, PP-system, Hitchin, United Kingdom) under constant leaf temperature (25°C) and CO_2_ concentration (390 mol⋅mol^−1^). The leaves were placed under 300 mol photon⋅m^−2^⋅s^−1^ for 15 min to fully activate the photosynthesis systems. The light series of photosynthetic light flux density were set as follows: 1600, 1200, 800, 600, 400, 200, 150, 100, 50, and 0 mol photon⋅m^−2^⋅s^−1^. All of the measurements were carried out from 9:00 to 11:00 am and 14:00 to 16:00 pm on sunny days. Three plants were measured each time.

The responses of net photosynthetic rate to different light levels were modeled using the non-rectangular hyperbola in the Farquhar model (Prioul and Chartier, 1977):

$$P_{n}(I)=\frac{\mathrm{AQY}*I+A_{\max}\sqrt{{\left(\mathrm{AQY}*I+A_{\max}\right)}^{2}-4\mathrm{AQY}*\theta *\text{I}*A_{\max}}}{2\theta }-R_{d}$$

where *P_n_* is the net photosynthetic rate (μmol⋅CO_2_ m^−2^⋅s^−1^), *A*_max_ is the light-saturated rate of CO_2_ accumulation (μmol⋅CO_2_ m^−2^⋅s^−1^), *I* is the photosynthetic photon flux density (μmol⋅m^−2^⋅s^−1^), *AQY* is the leaf maximum apparent quantum yield of CO_2_ uptake, θ is the convexity of the light response curve, and *R_d_* is the dark respiration (μmol⋅m^−2^⋅s^−1^). The quantum yield could be estimated from the initial slope by fitting linear regression to the low-photon flux data (less than 200 μmol⋅m^−2^⋅s^−1^) of the light response curve. The intersection point of the straight line with the *X*-axis corresponded to *LCP* (the light compensation point, μmol⋅m^−2^⋅s^−1^). The projection of *A*_max_ to the *X*-axis corresponded to *LSP* (the light saturation point, μmol⋅m^−2^⋅s^−1^).

### *A/C_i_* Curves

In the same leaves, the response of light-saturated CO_2_ assimilation to variable internal CO_2_ concentrations (*A*/*C_i_* curves) was measured as described in Guidi et al. (2008). *P_n_* was measured at CO_2_ concentrations of 50, 100, 200, 300, 360, 400, 600, 800, 1000, 1300, 1600, and 2000 mol⋅mol^−1^ at photosynthesis saturating irradiance 400 mol⋅photon⋅m^−2^⋅s^−1^. From the *A*/*C_i_* curves, the following photosynthetic parameters were calculated according to Long and Bernacchi (2003): maximum carboxylation (*V_cmax_*), maximum rate of RuBP regeneration (*J_max_*), and triose phosphate utilization (*TPU*). A buffer bottle was used to control the concentrations of CO_2_.

### Measurements of Leaf OJIP Transients

The fast Chl fluorescence induction kinetics curve was measured using a Handy-PEA chlorophyll fluorometer (Hansatech, United Kingdom). The leaves used to measure the net photosynthetic rate were dark-adapted for 30 min before determination of the minimal (*F*_0_) and maximal (*F_m_*) fluorescence yields. The fluorescence intensity was measured from 20 μs (initial fluorescence *F*_0_) to 1 s. Biolyzer HP3 software (Bioenergetics Lab., Geneva, Switzerland) was used to analyze the energy pipeline models and specific energy fluxes.

### Determination of Leaf Cd Content

Leaf samples were washed with deionized water and oven-dried at 105°C for 30 min and then 75°C until a constant weight was achieved and maintained. A ground sample of 0.250 g was microwave-digested (CEM, MARS5, United States) with 8 mL of HNO_3_ at 180°C for 30 min. Then, the leaf Cd concentration was determined by ICP-MS (Agilent ICP-MS 7700ce, Agilent Technologies, Santa Clara, CA, United States).

### Statistics Analysis

All of the parameters described above were measured 90 days after the plants were subjected to soil Cd treatments. SPSS 19.0 statistical software package (SPSS, Chicago, IL, United States) were used in the study to perform the statistical analyses. For each treatment, the mean values with standard deviation (±SD) are shown in the figures and tables. The parameters were analyzed by one-way analysis of variance (ANOVA, *p* < 0.05), followed by Duncan’s multiple range tests. The graphs were created using Origin 9.0 software (Origin Lab, United States).

## Results

### Cd Contents in Leaves

**FIGURE 1:**
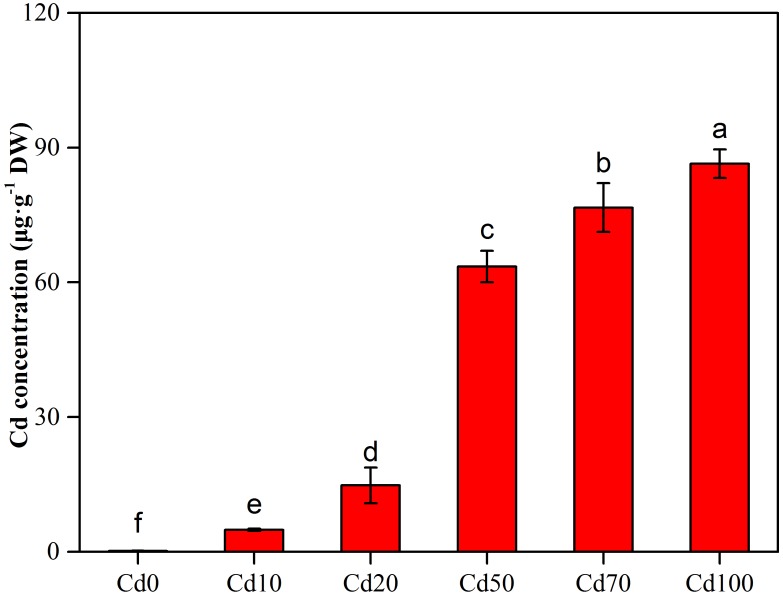
Effect of different concentrations of Cd concentration in leaves of *Hybrid Pennisetum*. Vertical bars represent ± SD of the mean (*n* = 3); different letters on the SD bars indicate significant differences among the Cd treatments (*p* < 0.05).

The Cd concentration in leaves of *Hybrid Pennisetum* exhibited remarkable differences among various Cd soil treatments (Figure 1). In the low soil Cd treatments (Cd10 and Cd20), the Cd concentration in leaves ranged from 4.9 to 15.8 μg g^−1^. With the increase in Cd added into the soil, the Cd concentration in leaves showed a sharp increase. At Cd100, the Cd concentration in leaves reaches 86.4 μg g^−1^, which is approximately 20 times higher than the Cd concentration in leaves from the Cd10 treatment.

### SPAD Values in Leaves

**FIGURE 2:**
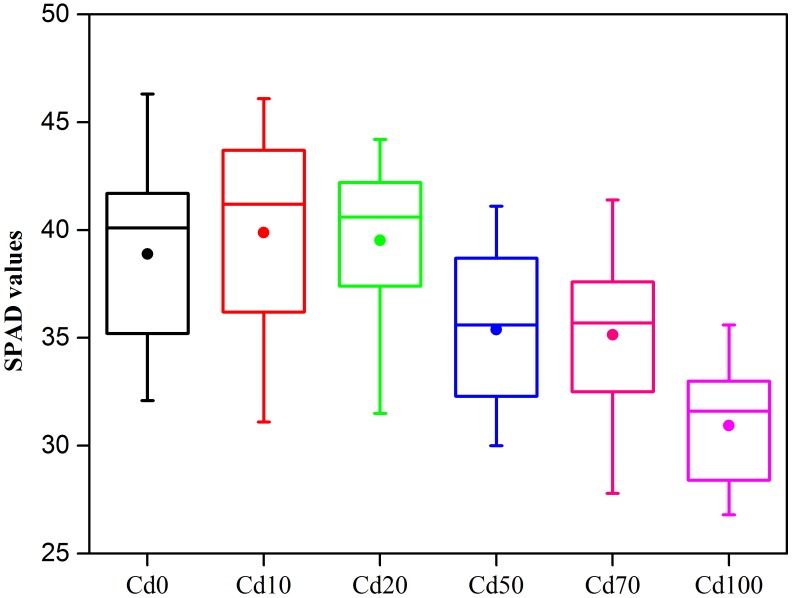
Boxplots of SPAD under different Cd stress. The solid circle in the box plots represents the mean values, *n* = 15.

The SPAD chlorophyll values of *Hybrid Pennisetum* are shown in Figure 2. Compared to Cd0, the chlorophyll values of the leaves in *Hybrid Pennisetum* were not influenced by 10 to 20 mg kg^−1^ of Cd^2+^ pollution. With the increase in Cd added to the soil, the SPAD chlorophyll values significantly decreased. Compared to Cd0 plants, the SPAD values of Cd50, Cd70, and Cd100 plants decreased by 9.0, 9.6, and 20.4%, respectively.

### Gas Exchange Parameters

**FIGURE 3:**
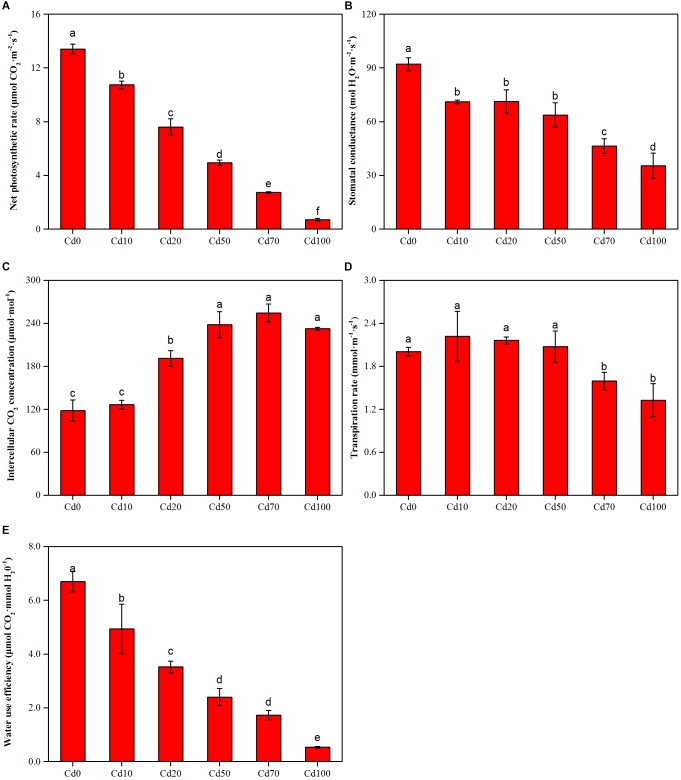
Net photosynthetic rate **(A)**, Stomatal conductance **(B)**, Intercellular CO_2_ concentration **(C)**, Transpiration rate **(D)**, and Water use efficiency **(E)** of *Hybrid Pennisetum* leaves treated with different doses of Cd concentration in soil. Vertical bars represent ± SD of the mean (*n* = 3); different letters on the SD bars indicate significant differences among the Cd treatments (p < 0.05).

Instantaneous gas exchange parameters corresponding to plants subjected to different soil Cd treatments are presented in Figure 3A–E. The maximum values of net CO_2_ assimilation rate (*P_n_*, Figure 3A), stomatal conductance (*G_s_*, Figure 3B), and water use efficiency (*WUE*, Figure 3E) found in Cd0 were 13.4 μmol⋅CO_2_ m^−2^⋅s^−1^, 92 mol⋅H_2_O m^−2^⋅s^−1^, and 6.7 μmol CO_2_ mmol⋅H_2_O^−1^, respectively. With the increase in soil Cd content, *P_n_*, *G_s_*, and *WUE* in leaves decreased significantly. The values of *P_n_*, *G_s_*, and *WUE* in Cd10, Cd20, Cd50, Cd70, and Cd100 were decreased from 10.7 to 0.7 μmol⋅CO_2_ m^−2^⋅s^−1^, 71.0 to 35.3 mol⋅H_2_O m^−2^⋅s^−1^, and 4.9 to 0.5 μmol⋅CO_2_ mmol⋅H_2_O^−1^, respectively. The intercellular CO_2_ concentration (*C_i_*, Figure 3C) in leaves showed the opposite trend as *P_n_*, *G_s_*, and *WUE*. *C_i_* in Cd0 and Cd10 had the lowest values: 118.3 and 126.3 μmol⋅mol^−1^, respectively. With the increase of soil Cd content, *C_i_* in the Cd20, Cd50, Cd70, and Cd100 treatments ranged from 191.0 to 254.3 μmol⋅mol^−1^. However, based on the one-way ANOVA, *C_i_* in high soil Cd content (Cd50, Cd70, and Cd100) plants showed no significant differences (*p* > 0.05, Figure 3C). The leaf transpiration rate (*T_r_*, Figure 3D) in Cd0 plants was 2.0 mmol⋅m^−1^⋅s^−1^. Although compared to Cd0, *C_i_* in Cd10, Cd20, and Cd50 plants increased 0.22, 0.17, and 0.07 mmol⋅m^−1^⋅s^−1^, respectively, there were no remarkable differences between them. With the increase in soil Cd content, *C_i_* in Cd70 and Cd100 plants showed a significant decrease, and the lowest value was 1.3 mmol⋅m^−1^⋅s^−1^ in Cd100 plants.

### Leaf Photosynthetic CO_2_ Response Parameters

**FIGURE 4:**
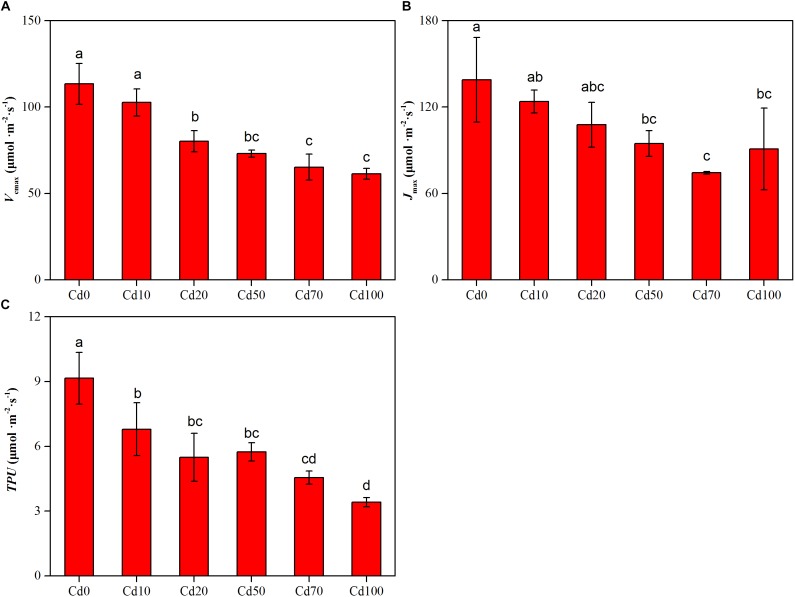
Effects of Cd treatment on the maximum velocity of Rubisco carboxylation **(A)**, the maximum velocity of RuBP regeneration **(B)**, and the triose phosphate utilization **(C)** in leaves of *Hybrid Pennisetum*. Vertical bars represent ± SD of the mean (*n* = 3); different letters on the SD bars indicate significant differences among the Cd treatments (*p* < 0.05).

The values for the maximum rate of Rubisco carboxylation (*V_cmax_*), the maximum rate of RuBP regeneration (*J_max_*), and the triose phosphate utilization (*TPU*) fitted from A/Ci curves are shown in Figure 4A–C. The changes in *V_cmax_*, *J_max_*, and *TPU* showed similar trends, decreasing with the enhancement of soil Cd content, and the highest values of *V_cmax_*, *J_max_*, and *TPU* were found in Cd0 plants (113.5, 138.9, and 8.2 μmol⋅m^−2^⋅s^−1^, respectively). Compared to Cd0 plants, *V_cmax_*, *J_max_*, and *TPU* in Cd10, Cd20, Cd50, Cd70, and Cd100 plants decreased by 10.8–52.1, 15.1–64.6, and 2.4–5.7 μmol⋅m^−2^⋅s^−1^, respectively.

### Light Response Curves

**FIGURE 5:**
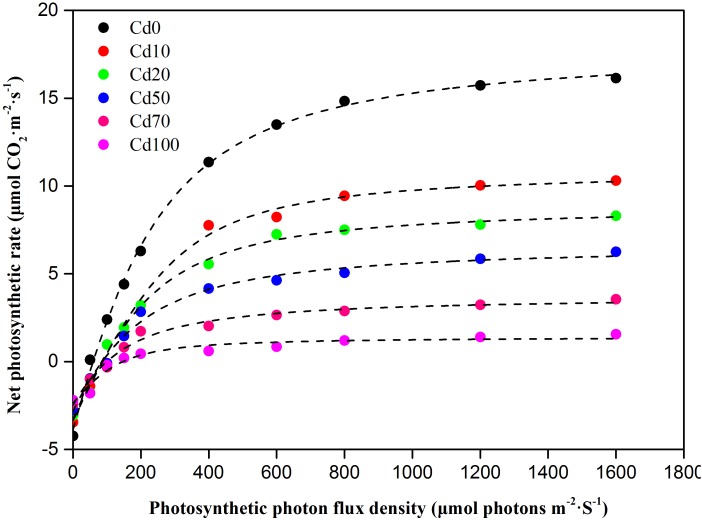
Photosynthetic light response curves in leaves of *Hybrid Pennisetum* under different soil Cd stress conditions.

The light response curves of *Hybrid Pennisetum* in various soil Cd treatments are illustrated in Figure 5. As shown in the light response curves, the response of *P_n_* to the light intensity is significantly different during different soil Cd treatments. The *P_n_* in leaves decreased with increasing Cd, and the differences between treatments were more obvious with increasing irradiance. When the level of PAR was higher than that of *LCP*, at the same PAR, the levels of *P_n_* were Cd0 > Cd10 > Cd20 > Cd50 > Cd70 > Cd100. According to the simulated analysis of the light response curves (Table 1), *A_max_*, *R_day_*, *AQY*, *LCP*, and *LSP* in the leaves of *Hybrid Pennisetum* under Cd0 treatment were 20.7 μmol⋅CO_2_ m^−2^⋅s^−1^, 3.7 μmol⋅m^−2^⋅s^−1^, 0.064 mol⋅mol^−1^, 64.6 μmol⋅m^−2^⋅s^−1^, and 473.7 μmol⋅m^−2^⋅s^−1^, respectively. When plants suffered soil Cd stress, *A_max_*, *R_day_*, *AQY*, and *LSP* in the leaves of *Hybrid Pennisetum* decreased with the increase in soil Cd, but *LCP* in leaves that grew with high soil Cd was higher than the *LCP* of leaves that grew with low soil Cd. Compared with the Cd0 treatment, *A_max_*, *R_day_*, and *AQY* in Cd10, Cd20, Cd50, Cd70, and Cd100 leaves decreased by 30.0–81.6, 5.4–35.1, and 29.7–62.5%, respectively, whereas *LCP* increased by 38.1–130.0%. Although compared to Cd0, the value of *LSP* increased by 17.7 μmol⋅m^−2^⋅s^−1^ in Cd10 leaves and decreased by 4.8 μmol⋅m^−2^⋅s^−1^ in Cd20 leaves, there were no significant differences among those three treatments (*p* < 0.05). When the soil Cd content reached 50 mg kg^−1^, *LSP* showed a remarkable decrease and had the lowest value (409 μmol⋅m^−2^⋅s^−1^) in the leaves of the Cd100 treatment.

**Table 1:** Cd stress effect on light-saturated photosynthesis, apparent quantum yield, light compensation point, light saturation point, and dark respiration in leaves of *Hybrid Pennisetum*.

Treatment	*A_max_* (μmol⋅CO_2_ m^−2^⋅s^−1^)	*R_day_* (μmol⋅m^−2^⋅s^−1^)	*AQY* (mol⋅mol^−1^)	*LCP* (μmol⋅m^−2^⋅s^−1^)	*LSP* (μmol⋅m^−2^⋅s^−1^)
Cd0	20.7 ± 0.3 a	3.7 ± 0.2 a	0.064 ± 0.004 a	64.6 ± 1.7 e	473.7 ± 8.2 a
Cd10	14.5 ± 0.2 b	3.5 ± 0.1 ab	0.045 ± 0.002 b	89.2 ± 1.6 d	491.4 ± 19.3 a
Cd20	12.2 ± 0.3 c	3.2 ± 0.2 b	0.042 ± 0.002 bc	92.2 ± 0.8 d	468.9 ± 36.4 ab
Cd50	9.3 ± 0.2 d	2.7 ± 0.1 c	0.037 ± 0.002 cd	96.5 ± 2.0 c	437.9 ± 10.5 bc
Cd70	6.2 ± 0.3 e	2.5 ± 0.3 c	0.034 ± 0.007 d	111.7 ± 2.7 b	419.0 ± 11.6 c
Cd100	3.8 ± 0.1 f	2.4 ± 0.01 c	0.024 ± 0.003 e	148.6 ± 2.1 a	409.4 ± 3.4 c

*Values are the mean of three replicates. Different letters indicate that values are significantly different from each other at p ≤ 0.05.*

### Chlorophyll *a* Fluorescence Transient Analysis

**FIGURE 6:**
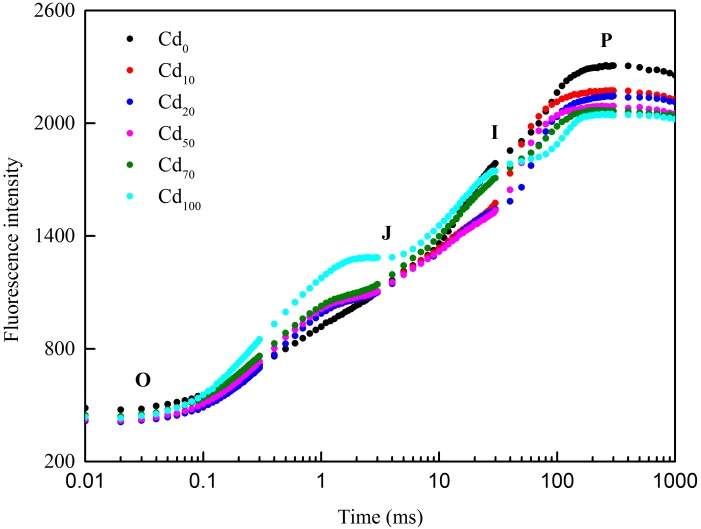
The fluorescence intensity of the original fluorescence kinetic curve of *Hybrid Pennisetum* leaves under different Cd treatments.

Chlorophyll *a* fluorescence induction kinetics was measured in order to evaluate the inhibitory effects of Cd on photochemical efficiency of PSII in *Hybrid Pennisetum* plants (Figure 6). The Chl *a* induction curves (O-J-I-P) obtained from different Cd treatments revealed that Cd stress caused a prominent change in the shape of fluorescence induction curve compared to control. At the O-J phase, a faster fluorescence increase was found in Cd-treated samples. With the reaction time go on, the curves from the I to P phase showed a significant decreased under Cd treatment.

**FIGURE 7:**
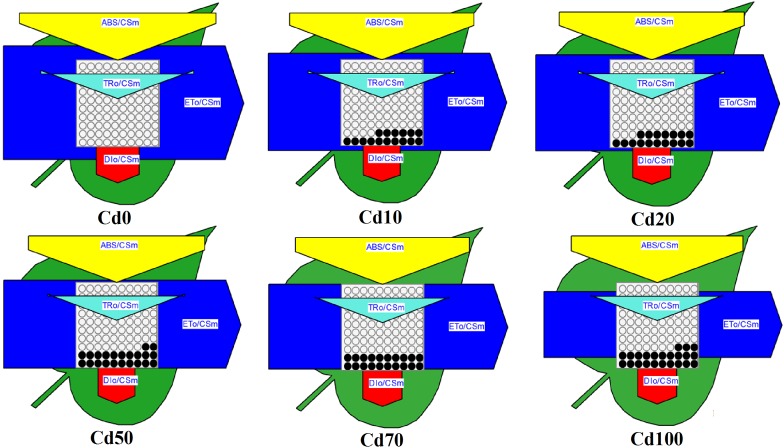
Energy pipeline leaf model of phenomenological fluxes (per cross-section, CS) in last fully expanded leaves as affected by Cd stress. Data are means ± standard errors (*n* = 3). Each relative value is drawn by the width of the corresponding arrow, standing for a parameter. Empty and full black circles indicate, respectively, the percentage of active (QA reducing) and non-active (non-QA reducing) reaction centers of PS II. TRO/CSm – trapped energy flux per CS; ETO/CSm – electron transport flux per CS; ABS/CSm – absorption flux per CS; DIO/CSm – non-photochemical quenching per CS.

A leaf model of phenomenological energy fluxes (per cross-section) was used in the study to present the alteration of PSII energy fluxes in response to Cd stress (Figure 7). In the dynamic model, the energy fluxes such as ABS/CSm, TRo/CSm, ETo/CSm, and DIo/CSm, which were expressed by the width of corresponding arrows, indicate the efficiency of light absorption, trapping and electron transport, and dissipation per cross-section of PS II in different Cd treatments. In the present study, there was no significant difference in energy absorbed per excited cross-section (ABS/CSm), trapped energy flux per CS (TRO/CSm), and non-photochemical quenching (DIO/CSm) under all Cd treatments. In contrast, the reoxidation of reduced QA via electron transport over a cross-section of active and inactive RCs (ETo/CSm, indicated by a significantly smaller blue arrow) was reduced significantly in *Hybrid Pennisetum* leaves under Cd stress and gradually decreased with increasing soil Cd content. Furthermore, the decreased density of the active RCs (indicated as open circles) and the increased density of the inactive RCs (expressed by open closed circles) in the PS II cross-section under Cd stress conditions suggest that electron transport was inhibited because 18–23% of active RCs were converted into inactive RCs under Cd stress conditions.

## Discussion

Phytoextraction is an important technique that has been widely used to remediate heavy metal contaminated soils by using accumulators to extract contaminants from soil into plants. An ideal phytoextraction plant should have a high metal bioaccumulation factor and a high metal tolerance (Zhao et al., 2003). In our study, there was significant Cd accumulation in the leaves of *Hybrid Pennisetum*, and the leaf Cd concentration exhibited a remarkable increase with the strength of soil Cd stress. The results indicated that *Hybrid Pennisetum* has a high ability to uptake Cd metal and quickly transfer Cd^2+^ from roots to leaves. According to the study of Lux et al. (2011), leaf Cd concentrations exceeding 5–10 μg g^−1^ are toxic to most plants. After 3 months of Cd treatments on *Hybrid Pennisetum*, the leaf Cd concentrations reached 63.5 μg g^−1^ at Cd50, 76.4 μg g^−1^ at Cd70, and 86.4 μg g^−1^ at Cd100 treatment. Although photosynthesis was extremely suppressed under high Cd treatments, *Hybrid Pennisetum* still had a low carbon assimilation ability (*P_n_* in Cd50, Cd70, and Cd100 were 4.9, 2.7, and 0.7 μmol CO_2_ m^−2^⋅s^−1^, respectively).

Chloroplasts are pigments that play an important role in light energy absorption, transmission, and conversion to chemical energy during photosynthesis. The reduction of chlorophyll content and the inhibition of chlorophyll synthesis were proved to be the primary causes of diminished photosynthetic activity (Yasemin et al., 2008). In the present study, the progressive decreased photosynthetic activity in *Hybrid Pennisetum* was partially attributable to the remarkably reduced chloroplast contents due to exposure to Cd (Figure 2). Similar results were observed in a previous study in which chlorophyll and carotenoid concentrations in leaves decreased as a result of Cd stress (Su and Wang, 2004; Pagliano et al., 2006; Ahammed et al., 2012; Xu et al., 2014; Per et al., 2016). The chloroplast reduction could be explained by Cd replacing the magnesium atom within chlorophyll to form a chlorophyll-Cd complex. This replacement results in damage to the photosynthetic apparatus (Siedlecka et al., 1998) and inhibits the production of chlorophyll by obstructing the formation of the protochlorophyllide reductase ternary complex and the synthesis of 5-aminolaevulinic acid (Huang et al., 1997) and by reducing chlorophyll synthesis (Gonçalves et al., 2009) or enhancing enzymatic degradation (Somashekaraiah et al., 1992).

**FIGURE 8:**
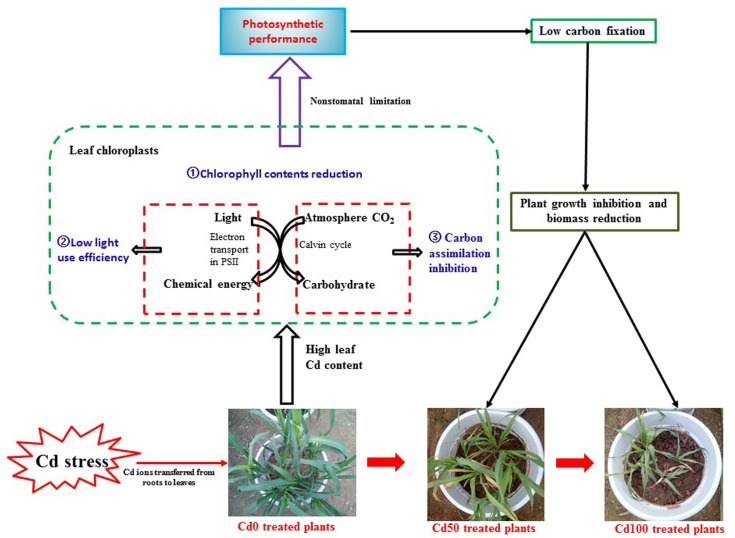
Schematic representation of the non-stomatal limitation in regulating the photosynthetic performance of *Hybrid Pennisetum* under Cd stress conditions. The figure shows that the decrease in leaf chlorophyll content, reduction of the ability to use light, and restrictions on carbon assimilation caused by high Cd content in leaves limit the capacity of *Hybrid Pennisetum* in carbon fixation.

Cadmium toxicity caused a notable reduction in the photosynthetic rate in different plant species (Alexander et al., 2008). However, in other studies, no direct Cd effects on the photosystem were found in either *Brassica juncea* (Haag et al., 1999) or *Arabidopsis thaliana* (Laetitia et al., 2010) plants. These contrasting results could be explained by a threshold of phytotoxic concentration of Cd toxicity in plants that depends on plant species, ecotypes, cultivars, and even plant tissues (He et al., 2015). In our study, Cd caused a significant reduction in *P_n_* (Figure 3A), indicating that plant carbon assimilation ability was suppressed due to Cd toxicity. Accompanied by the significant reduction in *P_n_*, lower *G_s_* (Figure 3B) and higher *C_i_* (Figure 3C) were simultaneously observed in *Hybrid Pennisetum* leaves. The results indicated that the decrease in *P_n_* did not result from a low *G_s_* or low CO_2_ concentration in chloroplasts because the *C_i_* levels were even higher in leaves of those plants treated with Cd than in the control, indicating that Cd reduced *P_n_* by reducing CO_2_ fixation by Rubisco (Wahid et al., 2010). Therefore, the inhibition of photosynthetic processes by Cd in *Hybrid Pennisetum* was due to non-stomatal restriction but not stomatal limitation. These findings agree with the effect of Cd shown on CO_2_ fixation in cucumber (Feng et al., 2010), lettuce (Monteiro et al., 2009), and *Ceratopteris pteridoides* (Deng et al., 2014). Although the stomata were closed by Cd stress, the *T_r_* of *Hybrid Pennisetum* in the Cd10, Cd20, and Cd50 treatments maintained a stable value (Figure 3D), indicating that the transpiration of *Hybrid Pennisetum* was not affected by the addition of 50 mg kg^−1^ Cd to the soil. On the contrary, *T_r_* in Cd70 and Cd100 treatment decreased significantly due to the remarkable stomata closure. Cd stress also produced disturbances in water balance, and a noticeable reduction of *WUE* was observed in all Cd treatments (Figure 3E). This could be explained by the inhibition of absorption and translocation of water, as previously observed by Barceló and Poschenrieder (1990) and Singh et al. (2008). Figure 8 exhibits the non-stomatal limitation in regulating the photosynthetic performance of *Hybrid Pennisetum* under Cd stress conditions.

**FIGURE 9:**
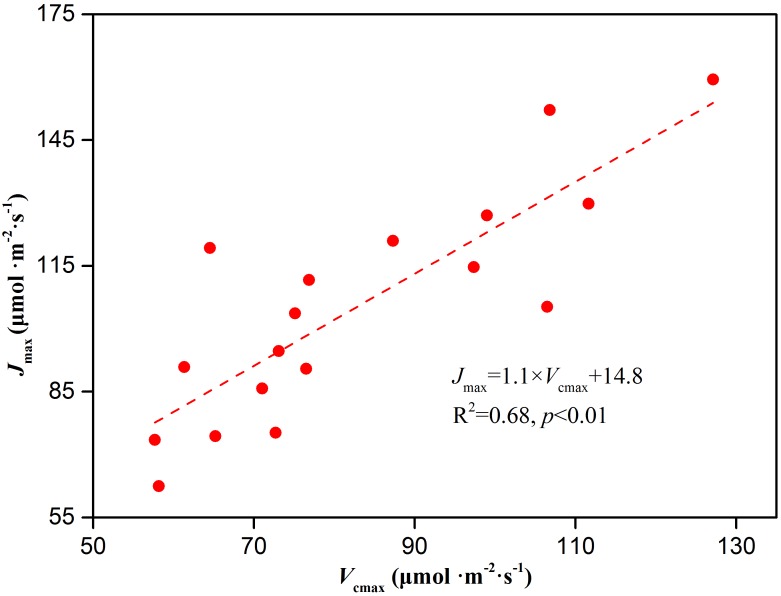
The relationship between *J_max_* and *V_cmax_* in leaves of *Hybrid Pennisetum*.

The non-stomatal limitation of photosynthesis could be expressed by the change in photosynthetic capacity. The *V_cmax_* at low CO_2_ concentrations and *J_max_* at high CO_2_ concentrations are often used to reflect the photosynthetic capacity of plants in response to environmental stress (Farquhar et al., 1980; Sharkey et al., 2007; Song et al., 2016). In our study, Cd stress caused a decrease in *V_cmax_* and *J_max_* of *Hybrid Pennisetum* (Figure 4). The decline in the *V_cmax_* might be due to the reduced amount of active Rubisco (Peña-Rojas et al., 2004), and the reduction in the *J_max_* might be due to the limited activity of photosynthetic enzyme (such as sedoheptulose-1,7-bisphosphatase and fructose-1,6-bisphosphatase) and the insufficient supply of NADPH or ATP (Lawlor and Cornic, 2010). The relationship between *V_cmax_* and *J_max_* represents resource allocation between the two photosynthetic cycles-electron transport and the Calvin–Benson cycle (Walker et al., 2015). In this study, *J_max_* was strongly related to *V_cmax_*, and neither leaf nitrogen, phosphorus, nor specific leaf area had a substantial impact on the relationship. Similar results were supported by the analysis of empirical data from 109 species (Wullschleger, 1993). As shown in Figure 9, although the changes in the two parameters were different among the various Cd treatments, there was a significant positive linear relationship between *J_max_* and *V_cmax_* (*p* < 0.01). The result suggested the viewpoint of (Walker et al., 2015), that tight coupling of *J_max_* with *V_cmax_* is a fundamental feature of plant photosynthetic trait relationships regardless of whether *Hybrid Pennisetum* suffered environmental stress. Triose phosphate utilization occurs when the chloroplast reactions have a higher rate than the capacity of the leaf to use the products of the chloroplasts (Sharkey et al., 2007). *TPU* is the rate of use of triose phosphates and it has been proposed to at least provide an indication of the feedback between growth and CO_2_ assimilation (Wullschleger, 1993). The drop in *TPU* in the Cd-treated plants (Figure 4) could be related to the decrease in the sink strength of the roots and leaves (Long and Bernacchi, 2003; Sharkey et al., 2007).

The light response curve has been widely used for reflecting the photochemical efficiency of plant photosynthesis in response to environmental factors such as drought (Song et al., 2016), elevated CO_2_ concentration (Herrick and Thomas, 1999), heat (Lewis et al., 1999), salt (Li and Ong, 1998), and metal stress (Zhang et al., 2014). The parameter *A*_max,_ which reflects the maximum photosynthetic capacity of the leaf (Zhou et al., 2010), is the maximum absolute value of photosynthesis under optimal environmental conditions (Tartachnyk and Blanke, 2004). In our study, *A_max_* was significantly reduced by Cd stress. As shown in Table 1, compared to Cd0, *A_max_* decreased by 6.2 μmol⋅CO_2_ m^−2^⋅s^−1^ even at a low Cd concentration (10 mg kg^−1^ in Cd10). *AQY* could be used to reflect the plant’s ability in absorbing, converting, and using light energy at low light intensities, and high *AQY* indicates that plants have a high light energy transfer efficiency (Marshall and Biscoe, 1980). In the present study, the change in *AQY* showed a similar trend as *A_max_* and decreased with increasing Cd concentration. This decline in the light use efficiency of *Hybrid Pennisetum* shows plants’ self-photoprotection mechanism in avoiding damage (Zhu et al., 2004). Under light saturation conditions, Cd stress inhibited the photochemical reactions, resulting the excitation energy could not be used in photochemical reactions totally. To protect the photosynthetic apparatus like chloroplast and cell membrane from being damaged by the excess excitation energy, the plant activates the self-photoprotection mechanism by dissipating the excess excitation energy as harmless heat through the xanthophyll cycle (Omasa and Takayama, 2003). With more heat dissipation, the light use efficiency was lower and the decrease in the photosynthetic rate was higher (Song et al., 2016).

**FIGURE 10:**
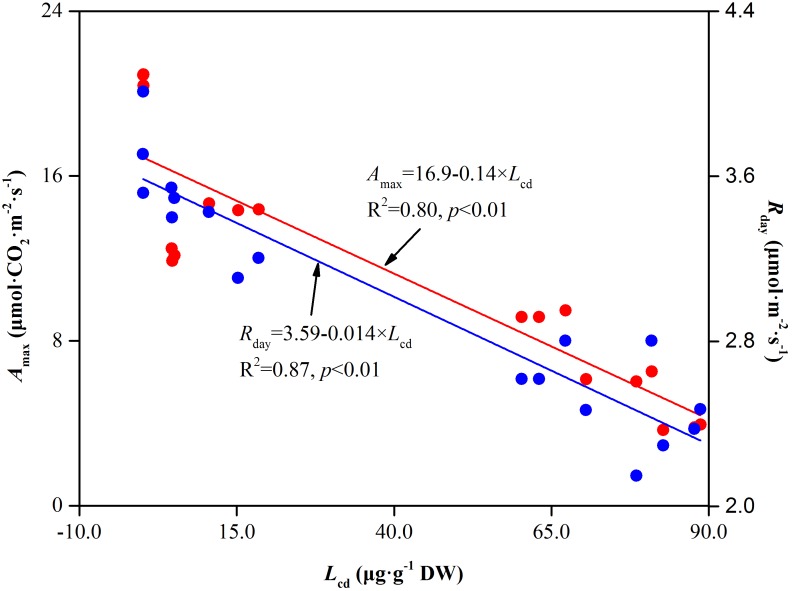
The relationship between leaf Cd concentration and respiration rate in leaves of *Hybrid Pennisetum*.

Light saturation point and *LCP* reflect the plant ability in using high and low light intensities. They are very useful primary indicators for measuring the relationship between light use and photosynthesis (Zhou et al., 2010). When the environmental conditions are not suitable, plants typically decrease the *LSP* or increase the *LCP* to ensure the normal operation of photosynthesis. In our study, compared to Cd0, *LSP* decreased and *LCP* increased with increasing soil Cd concentration (Figure 5 and Table 1), suggesting that soil Cd stress decreased the light use efficiency both at high light levels and at low light levels. The decreased light use efficiency at *LSP* showed the inhibition of CO_2_ assimilation ability in *Hybrid Pennisetum*. Furthermore, *LCP* is the light value at which the rate of CO_2_ release by respiration and photorespiration is equal to the rate of CO_2_ fixation by photosynthesis. High *LCP* reflects plants suffers higher respiration and lower Rubisco carboxylase activity or higher Rubisco oxygenase activity (Nunes et al., 2010). The obvious increase in *LCP* in Cd-treated plants observed in the present study implies that Cd could inhibit both photosynthesis and organic compounds accumulation in *Hybrid Pennisetum* (Zhang et al., 2014). *R_d_*, which is the index of the plant respiration rate in the dark, provides the power for a plant’s metabolic activity (Arnold et al., 2002). The decrease of *R_d_* under various Cd stress conditions, as shown in Table 1, reflects the reduction in the need for energy and intermediate metabolites, resulting in an extremely inhibited plant physiological activity (Atkin and Macherel, 2009). The Cd stress caused by the accumulation of Cd in leaves was the first and main reason for the reduction in both *A_max_* and *R_d_*. We hypothesized that there was a significant relationship between *A_max_* and *R_d_* and leaf Cd concentration (*L_cd_*). To test this hypothesis, linear curves were fitted to determine the relation between *A_max_* and *L_cd_* as well as the relation between *R_day_* and *L_cd_* in Figure 10. The fitted linear curves showed that *A_max_* and *R_day_* had a noticeable negative relationship with *L_cd_* (*p* < 0.01), indicating that the maximum photosynthetic capacity and respiration rate of *Hybrid Pennisetum* were significantly suppressed with the increase of Cd concentration in *Hybrid Pennisetum* leaves. It is important to note that we used *A_max_* but not *P_n_* to determine the relationship with *L_cd_*. The reason is that *P_n_* as an instantaneous measure parameter is very affected by the change in PPFD at different measurement times. Relatively, *A_max_* was more stable than *P_n_* because it was measured under optimal environmental conditions.

Chl fluorescence has a close relationship with plant photosynthesis, and Chl fluorescence analysis has been proven to be a useful tool in monitoring the influence of various stress conditions, such as salt (Mehta et al., 2010; Sun et al., 2016), high temperature (Mathur et al., 2015), pH (Tongra et al., 2011; Long et al., 2017), drought (Oukarroum et al., 2007), fluoranthene (Rupal and Anjana, 2013), photooxidation (Jing et al., 2017), and heavy metals (Hendrik et al., 2010; Goussi et al., 2018), on the photosynthetic activity of plants or microalgae/cyanobacteria. Although Chl fluorescence emissions only account for 2–5% of the energy that is not used in photosynthesis (Zhao et al., 2018), it can supply extensive information related to the components,structure, and function of plant photosynthetic performance (Strasser et al., 2004). Changes in Chl fluorescence indicate a decline in the ability of plants to cope with excess light intensity both in protective regulatory mechanisms and in the regulation of PSII (Logan, 2007). Therefore, any small change in Chl fluorescence in plants can be used to identify the response of plants to environmental stress. PSII of photosynthesis has been proven to be the primary target of Cd toxicity (Alexander et al., 2008; Popova et al., 2009). To characterize the PSII activities in Cd-treated plants, the OJIP transients that reflect the successive reduction in the electron pools (Pheo, QA and QB) of PSII (Strasser et al., 2004) were used in our study. The OJ phase in the OJIP transients is strongly light dependent (Neubauer and Schreiber, 1987; Schansker et al., 2015) and contains information on antenna size and connectivity between PSII reaction centers. The J to P rise in the OJIP transients is called the thermal phase and reflects a reduction in the rest of the electron transport chain (Schansker et al., 2005). As shown in Figure 6, a faster fluorescence increase in samples treated with different Cd stress was found at the OJ phase, indicating that the reoxidation of QA^−^ is inhibited and that there is a larger accumulation of QA^−^ due to the curtailment efficiency of electron transport beyond QA (Strasser and Govindjee, 1992; Strauss et al., 2006; Chen et al., 2011; Kalaji et al., 2017). With the increase in reaction times, the curves from the I phase to the P phase under Cd treatment decreased, indicating a slowdown in electron donation from the PSII donor side (Oukarroum et al., 2015). The different shapes of the fluorescence induction curves under different Cd treatments compared to the Cd0 treatment indicated that Cd toxicity not only inhibited the light use efficiency but also the ability to avoid photodamage.

The dynamic energy pipeline leaf model of the photosynthetic apparatus has been shown to be a direct and visual way to present the alteration of PSII energy fluxes (Li et al., 2014; Sun et al., 2016). In the present study, except for ETo/CSm, the other parameters, such as ABS/CSm, TR0/CSm, and DI0/CSm, showed no significant reduction during all Cd treatments compared with Cd0 plants (Figure 7). The unchanging ABS/CSm suggested that the energy trapped per excited CS was not suppressed by Cd. Electron transport (ETo/CSm) decreased with Cd treatment, indicating the inactivation of reaction center complexes. The ratio TR0/CSm and DI0/CSm was not significantly affected by Cd, indicating that the trapped light in the reaction centers was not affected significantly and that the excess energy was not dissipated in the form of heat but was utilized in some other form. In addition, the decreased ETo/CSm and relatively stable DIO/CSm contributed to the imbalance between light energy absorbed by photosystems and energy consumed by metabolic sinks in the plant.

## Conclusion

In this study, we concluded that the high Cd concentration in the leaves of *Hybrid Pennisetum* caused acute Cd toxicity by impairing photosynthetic performance after 3 months of soil Cd treatments. According to the changes in gas exchange parameters under Cd stress conditions, non-stomatal limitation, rather than stomatal limitation, regulates the photosynthetic processes of *Hybrid Pennisetum*. The mechanism by which Cd stress affected photosynthesis in *Hybrid Pennisetum* was through decreasing the leaf chlorophyll content, inhibiting Rubisco activity, RuBP regeneration, and triose phosphate utilization, reducing both light use and energy-providing ability, and preventing electron transport in PSII. The inhibition of photosynthetic processes caused a significant decrease in photosynthetic capacity.

## Data Availability

All datasets generated for this study are included in the manuscript.

## Author Contributions

WC and HJ conceived the experiment. XY, YH, XL, and XS conducted the experiment. XS analyzed the results and wrote the manuscript. All authors reviewed and approved the manuscript.

## Conflict of Interest Statement

The authors declare that the research was conducted in the absence of any commercial or financial relationships that could be construed as a potential conflict of interest.
